# Potential Hepatoprotective Role of Galectin-3 during HCV Infection in End-Stage Renal Disease Patients

**DOI:** 10.1155/2017/6275987

**Published:** 2017-04-11

**Authors:** Ruzica Lukic, Nevena Gajovic, Ivan Jovanovic, Milena Jurisevic, Zeljko Mijailovic, Veljko Maric, Biljana Popovska Jovicic, Nebojsa Arsenijevic

**Affiliations:** ^1^Department of Microbiology, Faculty of Medicine Foca, University of East Sarajevo, Sarajevo, Bosnia and Herzegovina; ^2^Center for Molecular Medicine and Stem Cell Research, Faculty of Medical Sciences, University of Kragujevac, Kragujevac, Serbia; ^3^Department of Infectology, Faculty of Medical Sciences, University of Kragujevac, Kragujevac, Serbia; ^4^Department of Surgery, Faculty of Medicine Foca, University of East Sarajevo, Sarajevo, Bosnia and Herzegovina

## Abstract

Hepatitis C virus infection (HCV), one of the greatest causes of liver disease, is a frequent complication in patients with end-stage renal disease (ESRD) on dialysis. ESRD is defined as decreased glomerular filtration and also accompanied by impaired function of the immune system. Galectin-3 is a *β*-galactoside-binding lectin, involved in various biological processes including pathogenesis of chronic renal disease. The aim of our study was to estimate disease severity in ESRD HCV^+^ patients and analyze the serum concentrations of IL-1*β*, IL-4, IL-23, and IL-6; anti-HCV antibodies; and galectin-3. Also, we attempted to determine potential correlation between galectin-3 level and parameters of disease severity ALT and AST. Our results showed decreased levels of ALT and AST (*p* = 0.00), demonstrating less liver destruction in ESRD HCV^+^ patients in comparison to HCV^+^ patients. Increased levels of IL-6 (*p* = 0.03) implicate a hepatoprotective role of IL-6 in these patients. Also, level of galectin-3 (*p* = 0.00) in the serum of ESRD HCV^+^ patients was higher than that of HCV^+^ patients. This alteration was accompanied with negative correlation between galectin-3 and AST and ALT, respectively (*p* = 0.029; *p* = 0.033). The presence of increased systemic levels of IL-6 and Gal-3 in ESRD HCV^+^ patients may be an attempt to counteract or limit ongoing proinflammatory processes and to downregulate chronic inflammation, suggesting the new aspects of HCV infection in ESRD patients.

## 1. Introduction

Hepatitis C virus (HCV) infection is one of the greatest causes of liver disease and a major risk factor for development of cirrhosis and hepatocellular carcinoma [1]. Recent epidemiological studies have revealed that more than 100 million persons have diagnosed HCV infection worldwide [2]. HCV does not have the ability to directly destroy hepatocytes; however, it activates host's innate and acquired immune system thus accelerating liver injury [3]. Once it enters in the hepatocyte, HCV uses different mechanisms for antigene changes and avoids host's immune response thus stimulating the development of chronic infection in the liver [4]. Although antivirus-acquired immune response includes activation of cellular and humoral components, it is well known that cellular immune response has a predominant role in the elimination of HCV-infected hepatocytes [5].

End-stage renal disease (ESRD) represents one of the greatest worldwide health issues [6]. Although there are differences in incidence and prevalence based on country, recent studies placed ESRD as the 18th factor of death [7]. Earlier studies have confirmed the importance of diabetes mellitus and cigarette smoking as main risk factors for ESRD development [8]. ESRD is defined as decreased glomerular filtration and albuminuria and is subdivided into five stages based on the level of urinary protein excretion and renal function [9]. It is one of the important causes for cardiovascular disease and mortality and reduced life quality [10]. ESRD is accompanied by inflammation and impaired function of the immune system [11]. Immune deficiency is reflected by decreased phagocytic and antigen-presenting cell function and impaired humoral and cellular immune response due to depletion of B lymphocytes as well as naive and memory CD4^+^ and CD8^+^ T lymphocytes [12].

Hepatitis C virus infection is one of the major complications in patients with ESRD on dialysis [13]. In spite of paying more attention on this group of patients, the annual incidence of hepatitis C infection in patients with end-stage renal disease is 100–1000 times higher in comparison to that in nondialyzed patients and varies in the range from 0.2% to 6.2% [14, 15]. Exposure to blood and blood products, internal contamination of hemodialysis machines, nosocomial spreading, and long dialysis duration are the main routes of HCV transmission in the ESRD patients [16, 17]. In many cases, HCV infection in ESRD patients does not produce symptoms and clinical manifestations which are accompanied with normal level of serum aminotransferase and gamma-glutamyltransferase [18]. Moreover, recent studies have noticed less progression of cirrhosis and hepatocellular carcinoma in the group of HCV + ESRD patients in comparison to HCV^+^ patients [19, 20]. Mechanisms underlying this phenomenon remain elucidated.

Galectin-3 is a multifunctional *β*-galactoside-binding lectin expressed in different cells such as epithelial and endothelial cells and leukocytes [21]. Different position of galectin-3, in nucleus, cytoplasm, or on the cell surface, enables its interaction with extracellular and intracellular ligands thus explaining a multifunctional role of this molecule in cell biology [22, 23]. Galectin-3 is involved in several biological processes including cell proliferation, differentiation, and apoptosis and also plays an important role in regulation of host immune response in inflammation, infection, and cancer [19, 21, 23].

Earlier studies revealed the importance of Gal-3 as a prognostic marker for different hepatic diseases. It is shown that systemic level of Gal-3 is significantly decreased in patients with cirrhosis and hepatocellular cancer compared to patients with chronic HCV infection [24]. It is also known that there is a strong correlation between increased systemic level of Gal-3 and both renal dysfunction and higher risk for chronic renal disease development [25]. However, the role of galectin-3 in biology of ESRD patients infected with hepatitis C virus still remains unclear.

The aim of this study was to estimate disease severity in ESRD HCV^+^ patients. We analyzed serum level of AST, ALT, and LDH, as well as serum concentrations of IL-1*β*, IL-4, IL-23, and IL-6; anti-HCV antibodies; and galectin-3. We report that ESRD HCV^+^ patients have less liver destruction based on lower serum level of markers AST, ALT, and LDH. We also demonstrate that serum levels of proinflammatory cytokines IL-1*β* and IL-23 as well as IL-4 do not differ among defined groups. However, the level of hepatoprotective IL-6 was higher in the serum of ESRD HCV^+^ patients. We also note increased serum level of galectin-3 and moderate negative correlation between galectin-3 and AST and between galectin-3 and ALT.

Our findings reveal a potentially hepatoprotective role for galectin-3 during HCV infection in ESRD patients.

## 2. Material and Methods

### 2.1. Ethical Approvals

The study was conducted at the University Hospital of Foca, Bosnia and Herzegovina, University Medical Center, Kragujevac, Serbia, and Center for Molecular Medicine and Stem Cell Research, Faculty of Medical Sciences, University of Kragujevac, Serbia. All patients gave their informed consent. Ethical approvals were obtained from relevant Ethics Committees of the University Hospital of Foca, Bosnia and Herzegovina, University Medical Center, Kragujevac, Serbia, and Faculty of Medical Sciences, University of Kragujevac, Serbia. All research procedures were made according to the Principle of Good Clinical Practice and the Declaration of Helsinki.

### 2.2. Patients

Study included three experimental groups with 40 patients with end-stage renal disease (ESRD) and hepatitis C viral infection (HCV), 20 hepatitis C-positive patients, and 20 patients with end-stage renal disease. Control subjects (normals (Nm)) were selected from volunteer blood donors at the University Hospital of Foca, Bosnia and Herzegovina. A control group consisted of 20 healthy individuals and was matched with the experimental groups on the basis of gender.

### 2.3. Evaluation of Biochemical Parameters in Sera

Serum levels of urea, creatinine, aspartate aminotransferase (AST), alanine aminotransferase (ALT), and lactate dehydrogenase (LDH) were routinely determined by standard methods suggested by the *IFCC* (International Federation for Clinical Chemistry and Laboratory Medicine) in the Central Biochemical Laboratory of the University Hospital of Foca, Bosnia and Herzegovina.

### 2.4. Measurement of HCV RNA

Quantitative measurements of serum HCV RNA in patients with chronic hepatitis C were performed using a real-time PCR commercial kit for quantification (Cobas Amplicor HCV monitor test, version 2.0, Roche Diagnostics Systems, Mannheim, Germany) according to the manufacturer's instructions with analytic sensitivity from 25 IU/ml to more than 100,000,000 IU/ml.

### 2.5. Measurement of Cytokines

The blood samples of ESRD and HCV^+^ patients as well as ESRD patients were collected before dialysis. The control group consisted of 20 HCV^+^ patients and 20 healthy individuals. Control subjects were selected from volunteer blood donors at the University Hospital of Foca. A control group was matched with the experimental groups on the basis of gender. Blood samples were collected from each studied subject; blood clot was cut and centrifuged for separating the serum and all serum samples were kept at −20 °C before use. Serum concentrations of cytokines were measured, as described [26] using sensitive enzyme-linked immunosorbent assay (ELISA) kits (R&D Systems, Minneapolis, MN, for IL-1*β*, IL-23, IL-4, and IL-6; measurement of cytokines according to the manufacturer's instructions).

### 2.6. Measurement of Anti-HCV Antibodies

Serological diagnosis of HCV infection was based on qualitative detection of anti-HCV antibodies in sera of patients by CMIA (chemiluminescent microparticle immunoassay). All patients were subjected to conformational test, western blot assay, MIKROGEN Diagnostic. This method includes detecting of antibodies specific for antigen core 1 and core 2, helicase, NS3, NS4, and NS5.

### 2.7. Statistical Analysis

The statistical analyses were performed using SPSS 20.0 software. The results were reported as mean, standard deviation (SD), and standard error (SE). In determining statistically significant difference between the means of two groups, Student's *t*-test was used for independent samples if the data had normal distribution or Mann–Whitney *U* test for data without normal distribution. Pearson's correlation evaluated the possible relationship between the cytokines and liver damage markers. Strength of correlation was defined as negative or positive and weak (−0.3 to −0.1 or 0.1 to 0.3), moderate (−0.5 to −0.3 or 0.3 to 0.5), or strong (−1.0 to −0.5 or 1.0 to 0.5). *p* value of 0.05 was considered statistically significant.

## 3. Results

### 3.1. ESRD Diminishes Liver Destruction in HCV

Forty ESRD and HCV^+^ patients, twenty HCV^+^, twenty ESRD, and twenty healthy controls (Nm) were enrolled in the study. Clinical and pathologic characteristics of these patients are presented in Table 1. There was no significant difference in gender distribution between defined groups of patients. Patients were similar in age, mean age 55.2 ± 9.32 in the ESRD HCV^+^ patient group versus 53.8 ± 10.38 in the HCV^+^ patient group versus 55.6 ± 12.72 in the ESRD group versus 52.9 ± 7.49 in the healthy control group of patients. Significant difference was observed in duration of dialysis between ESRD HCV^+^ patients (13.8 ± 8) and ESRD patients (3.85 ± 1.53) (*p* = 0.00). There was no statistically significant difference in the duration of HCV infection between ESRD HCV^+^ group of patients (7.02 ± 4.28) and HCV^+^ patients (8.25 ± 4.68).

**Figure 1:**
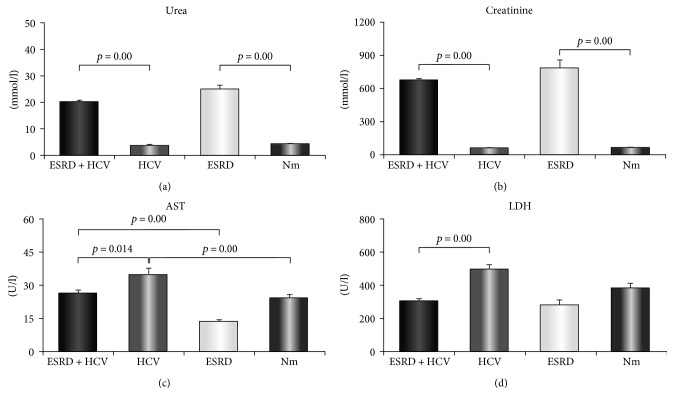
Serum values of biochemical parameters. Patients were divided into four groups: 40 patients with end-stage renal disease and hepatitis C viral infection (ESRD HCV^+^), 20 hepatitis C-positive patients (HCV^+^), 20 patients with end-stage renal disease (ESRD), and 20 healthy control individuals—normals (Nm). (a, b) Increased concentration of urea and creatinine in ESRD patients compared to healthy control as well as in ESRD HCV^+^ patients compared to HCV^+^ patients. (c) Significantly decreased concentration of aspartate aminotransferase (AST) in ESRD HCV^+^ patients in comparison to HCV^+^ patients. (d) Significantly decreased level of LDH in sera of ESRD HCV^+^ patients compared to HCV^+^ patients. Statistical significance was tested by independent samples *t*-test or Mann–Whitney rank sum test, where appropriate.

We have compared serum concentrations of urea and creatinine in all defined groups. Mean concentrations of urea and creatinine were significantly higher in ESRD patients compared to healthy control (mean ± standard error 24.77 ± 1.71 versus 4.28 ± 0.22 mmol/l, *p* = 0.00, Figure 1(a); 777.96 ± 80 versus 63.68 ± 1.87 mmol/l, *p* = 0.00, Figure 1(b)). Our results also showed that serum levels of urea and creatinine were significantly higher in ESRD HCV^+^ patients compared to HCV^+^ patients (19.90 ± 0.91 versus 3.77 ± 0.34 mmol/l, *p* = 0.00, Figure 1(a); 667.11 ± 21.74 versus 57.07 ± 1.61 mmol/l, *p* = 0.00, Figure 1(b)).

Further, we measured liver function markers, and our results showed that ESRD HCV^+^ patients had significantly lower serum concentration of aspartate aminotransferase (AST) (26.10 ± 1.71 versus 34.53 ± 3.22 U/l; *p* = 0.014, Figure 1(c)) and decreased serum level of alanine aminotransferase (ALT) (it did not reach statistical significance, data not shown), compared to HCV^+^ patients. We also measured lactate dehydrogenase (LDH) in the sera of all defined groups. ESRD HCV^+^ patients had significantly decreased level of LDH in comparison to HCV^+^ patients (300.40 ± 18.40 versus 495.88 ± 27.79 U/l; *p* = 0.00, Figure 1(d)).

### 3.2. Hepatoprotective IL-6 Increases in ESRD HCV^+^ Patients

**Figure 2:**
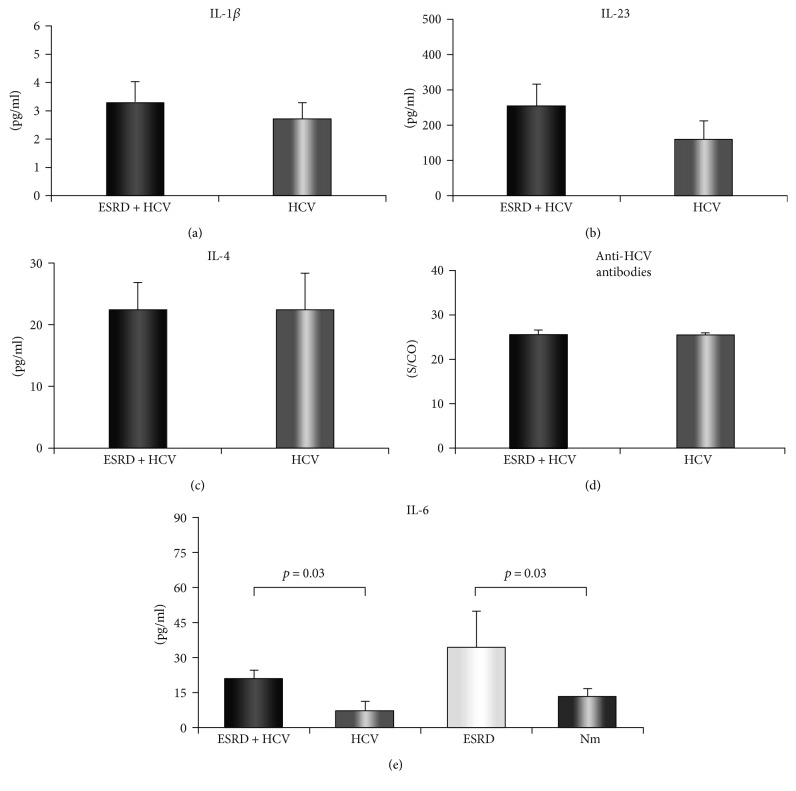
Serum values of IL-6 and anti-HCV antibodies. Patients were divided into four groups, based on kidney function and hepatitis C viral infection. Serum levels of cytokines were determined by ELISA. (a, b, c) There were no significant difference in serum levels of IL-1*β*, IL-23, and IL-4. (d) Anti-HCV antibodies were measured in sera of ESRD HCV^+^ and HCV^+^ patients. Significant difference was not observed in the level of anti-HCV antibodies in ESRD HCV^+^ patients compared to HCV^+^ patients. Anti-HCV antibodies were determined by ELISA. (e) Increased serum concentration of IL-6 in ESRD patients compared to the healthy control as well as in ESRD HCV^+^ patients compared to HCV^+^ patients.

Next, we examined the serum concentrations of cytokines IL-1*β*, IL-4, IL-23, and IL-6. Comparison of IL-1*β*, IL-23, and IL-4 serum levels did not reveal statistically significant difference between groups of ESRD HCV^+^ patients and HCV^+^ patients (IL-1*β*: 3.32 ± 0.72 versus 2.71 ± 0.57 pg/ml, *p* > 0.05, Figure 2(a); IL-23: 253.58 ± 62.93 versus 157.61 ± 54.69 pg/ml, *p* > 0.05, Figure 2(b); and IL-4: 22.36 ± 4.47 versus 22.42 ± 5.93 pg/ml, *p* > 0.05, Figure 2(c)). We have also measured serum level of anti-HCV antibodies. The result showed that there was no significant difference in the serum level of anti-HCV antibodies in ESRD HCV^+^ patients compared to HCV^+^ patients (25.49 ± 1.11 versus 25.47 ± 0.49 S/CO; *p* > 0.05, Figure 2(d)).

Further, we analyzed concentration of IL-6 in serum of all four defined groups. ESRD patients had significantly increased level of IL-6 compared to the healthy control (34.46 ± 15.43 versus 13.56 ± 3.1 pg/ml; *p* = 0.03, Figure 2(e)). Serum level of IL-6 was also significantly increased in the group of ESRD HCV^+^ patients compared to HCV^+^ patients (20.79 ± 3.81 versus 7.36 ± 3.87 pg/ml; *p* = 0.03, Figure 2(e)).

### 3.3. Sera of ESRD Patients Contain Higher Galectin-3 Level

**Figure 3:**
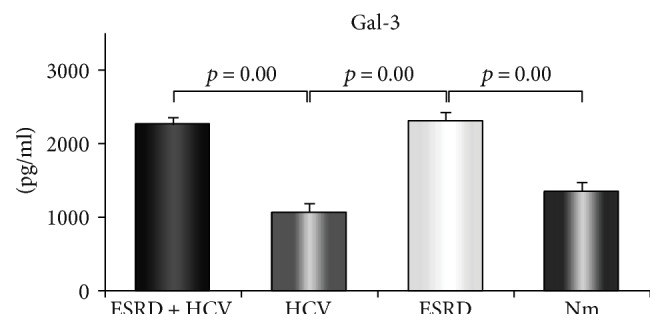
Serum values of galectin-3 and ratio of galectin-3 and proinflammatory and immunosupressive cytokines. Galectin-3 was analyzed in all four groups of patients. Increased level of galectin-3 in ESRD patients compared to the healthy control as well as significantly increased level of galectin-3 in ESRD HCV^+^ patients in comparison to HCV^+^ patients. Serum level of galectin-3 was determined by ELISA.

We have measured serum levels of galectin-3 in all four groups of patients. ESRD patients had significantly increased concentration of galectin-3 compared to healthy control (2308.31 ± 111.29 versus 1341.90 ± 127.85 pg/ml; *p* = 0.00, Figure 3). Our results also showed that ESRD HCV^+^ patients had significantly increased level of galectin-3 in comparison to HCV^+^ patients (2252.50 ± 97.18 versus 1072.76 ± 108.80 pg/ml; *p* = 0.00, Figure 3).

**Figure 4:**
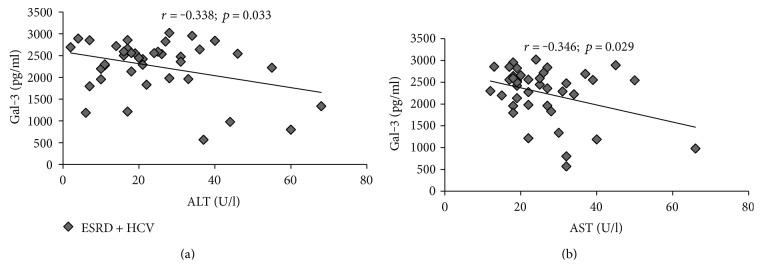
Galectin-3 and liver damage marker correlation. Correlations were analyzed between galectin-3 and markers of liver damage in the group of patients with end-stage renal disease and hepatitis C viral infection. Based on *r* value, correlation can be positive or negative and weak (*r* = 0.1–0.3), moderate (*r* = 0.3–0.5), and strong (*r* > 0.5). (a, b) Moderate negative correlation was obtained between galectin-3 and AST as well as galectin-3 and ALT in ESRD HCV^+^ patients.

We also assessed correlations between galectin-3 and liver damage markers in ESRD HCV^+^ patients. There is moderate negative correlation between galectin-3 and AST (*r* = −0.346; *p* = 0.029; Figure 4(a)) as well as between galectin-3 and ALT (*r* = −0.338; *p* = 0.033; Figure 4(b)) in ESRD HCV^+^ patients.

## 4. Discussion

In the present study, we showed decreased level of AST and LDH in ESRD HCV^+^ patients compared to HCV^+^ patients. Higher systemic value of IL-6 was detected in ESRD HCV^+^ patients in comparison to HCV^+^ patients while there were no significant differences in the concentrations of IL-1*β*, IL-4, and IL-23 and anti-HCV antibodies between defined groups. For the first time, we have showed that galectin-3 was significantly increased in the sera of ESRD HCV^+^ patients in comparison to HCV^+^ patients. We observed moderate negative correlation between galectin-3 and AST as well as between galectin-3 and ALT in ESRD HCV^+^ patients.

Hepatitis C virus infection is one of the greatest causes of liver disease [27]. In order to predict or evaluate the level of liver damage, many studies have shown the importance of measuring AST and ALT during HCV infection [28, 29]. All these studies suggest on the importance of time of sample collection. In our study, blood samples of ESRD and HCV^+^ patients as well as ESRD patients were collected before dialysis, so a time factor could not affect the level of AST and ALT. We found decreased serum levels of AST, LDH (Figures 1(c) and 1(d)), and ALT (did not reach statistical significance, data not shown) in ESRD HCV^+^ patients in comparison to the HCV^+^ group. These results may suggest milder liver destruction of ESRD HCV^+^ patients compared to HCV^+^ patients. Our results are in line with majority of paper studying liver damage suggesting that predominantly serum level of AST correlates with markers of hepatic inflammation and fibrosis and thus can be an objective marker for evaluation of liver damage [29].

Many studies have shown that ESRD is accompanied with immune dysregulation by changing effector functions of innate and adaptive immunity which increases susceptibility to infections [30–32]. Presented results implicate that decreased function of immune system in ESRD patients leads to reduced elimination of virus-infected cells in comparison to HCV^+^ patients without ESRD. Our next goal was to investigate serum level of cytokines. Results showed that there was no significant difference in serum concentration of IL-1*β* and IL-23 between ESRD HCV^+^ patients and HCV^+^ patients (Figures 2(a) and 2(b)). As IL-1*β* and IL-23 are proinflammatory cytokines produced by cells of innate immunity [33], we believe there is no significant alteration in innate anti-HCV immune response in ESRD HCV^+^ patients compared to HCV^+^ patients.

Besides the significance of cellular immune response in HCV infection, humoral immunity is also important in combating viral infections [34]. It is well known that IL-4 is one of the critical cytokines for the development of humoral immune response [35]. Our results have shown that there is no difference in concentration of IL-4 in the serum of ESRD HCV^+^ and HCV^+^ patients (Figure 2(c)). Moreover, we did not find difference in serum concentration of anti-HCV antibodies between ESRD HCV^+^ patients and HCV patients (Figure 2(d)). These findings are in line with the results from another study revealing that there are no differences in serum level of IgG, IgM, and IgA in ESRD patients compared to the healthy control indicating that ESRD did not affect humoral arm of antiviral immune response. Some studies did show that renal disease affects components of cellular immune response [36]; however, we did not find difference in systemic levels of cytokines involved in cellular immune response between ESRD HCV^+^ and HCV^+^ patients. Since we did not find alterations in parameters of cellular and humoral immune response, we believe that some other mechanisms are responsible for less liver destruction.

In order to reveal possible mechanism for reduced liver damage in ESRD patients, we measured systemic level of IL-6 in ESRD HCV^+^ and HCV^+^ patients. IL-6 is known as hepatoprotective cytokine important for stimulating proliferation of hepatocytes as well as nonparenchymal cells in the liver [37]. During infection, IL-6 not only plays a major role in induction of an acute phase protein production in the liver but also facilitates expression of many protective genes in the hepatocytes thus stimulating hepatocyte proliferation and their survival [38–40]. The role of IL-6 in biology of HCV infection has not been elucidated yet. Although there are results showing that based on type of disease, IL-6 may act as proinflammatory or anti-inflammatory cytokine [41], we believe that IL-6 has protective role in ESRD HCV^+^-infected patients. Namely, we found significantly higher systemic level of IL-6 in ESRD HCV^+^ patients in comparison to HCV^+^ patients (Figure 3). Further, we noticed higher serum level of IL-6 in ESRD patients in comparison to the healthy control group (Figure 3), indicating that increment of IL-6 in ESRD HCV^+^ patients is probably due to renal failure. In line with previously described mechanisms of action, we believe that increased systemic level of IL-6 in ESRD HCV^+^ patients protects the liver from virus destruction subsequently decreasing systemic level of AST and ALT and also stimulates liver regeneration.

As earlier studies revealed potential mechanisms of action of Gal-3 as an anti-inflammatory and tissue protective marker, it was of interest to study it in ESRD HCV infection comorbidity. We found increased level of Gal-3 in the group of ESRD HCV^+^ patients compared to HCV^+^ patients, as well as in ESRD patients compared to the healthy control (Nm) (Figure 3). Also, we try to determine potential correlations between serum levels of Gal-3 and liver damage markers, such as ALT and AST. We showed negative correlation between galectin-3 and AST and ALT, respectively, in ESRD HCV^+^ patients (Figures 4(a) and 4(b)). During HCV infection, virus-coded structural as well as nonstructural proteins induce hepatocyte apoptosis and subsequent liver damage [42]. The antiapoptotic role of Gal-3 is well established. It has been shown that Gal-3 protects mitochondrial membrane integrity, inhibits releasing of cytochrome C, and thus keeps cells from programmed cell death [43]. The protective role is characteristic of intracellular Gal-3. A recent study described uptake of extracellular Gal-3 and accumulation in cytoplasm [44]. Taken together, Gal-3 could pass hepatocyte membrane and stabilize mitochondrial membrane, thus inhibiting caspase activation and preventing apoptosis and liver damage. Based on these findings, we believe that Gal-3 act hepatoprotective in ESRD HCV^+^ patients through diminishing of apoptosis. This phenomenon is reflected by lower serum level of AST and ALT in comparison to HCV^+^ patients and negative correlation between galectin-3 and AST and ALT, respectively.

In summary, we provide evidence for milder liver destruction in ESRD HCV^+^ patients compared to HCV^+^ patients. Increased systemic level of IL-6 and Gal-3 estimated in ESRD HCV^+^ patients in comparison to HCV^+^ patients may be present as a way to counteract or limit ongoing proinflammatory processes and prevent the liver from virus destruction. Our findings point on the yet unrecognized protective role of Gal-3 during HCV infection in ESRD patients.

## Figures and Tables

**Table 1:** Demographic data and clinical measures.

Parameter	ESRD and HCV	HCV^+^	ESRD	HC
Gender				
Men	18	9	13	12
Women	22	11	7	8
Age (years ± SE)	55.2 ± 1.47	53.8 ± 2.32	55.6 ± 2.84	52.9 ± 1.67
Dialysis (years ± SE)	13.8 ± 1.26^∗^	—	3.85 ± 0.34^∗^	—
HCV status (years ± SE)	7.02 ± 0.67	8.25 ± 1.04	—	—

^∗^ *p* < 0.05 (Mann–Whitney test).
